# Mechanical Behavior Monitoring and Load Inversion Analysis of Large-Diameter Underwater Shield Tunnel during Construction

**DOI:** 10.3390/s24041310

**Published:** 2024-02-18

**Authors:** Si-Yuan Ma, Xiao-Wei Ye, Zhi-Xiong Liu, Yang Ding, Di Zhang, Feng Sun

**Affiliations:** 1 Department of Civil Engineering, Zhejiang University, Hangzhou 310058, China; cesyma@zju.edu.cn (S.-Y.M.); cexwye@zju.edu.cn (X.-W.Y.); liuzhix@zju.edu.cn (Z.-X.L.); 2 Department of Civil Engineering, Hangzhou City University, Hangzhou 310015, China; 3 China Railway Siyuan Survey and Design Group Co., Ltd., Wuhan 430063, China; zdiok@126.com (D.Z.); sunfeng5433@126.com (F.S.)

**Keywords:** Structural Health Monitoring, shield tunnel, Fiber Bragg grating, inversion analysis, Parameter estimation, optimization algorithm

## Abstract

The construction of large-diameter shield tunnels underwater involves complex variations in water and earth load outside the tunnel segment, as well as intricate mechanical responses. This study analyzes the variation laws of external loads, axial forces, and bending moments acting on the segment ring during the shield assembly and removal from the shield tail. It accomplishes this through the establishment of an on-site monitoring system based on the Internet of Things (IoT) and proposes a Bayesian-genetic algorithm model to estimate the water and earth pressure. The fluctuation section exhibits a peak load twice as high as that in the stable section. These variations are influenced by Jack thrust, shield shell force, and grouting pressure. The peak load observed in the fluctuation section is twice as high as the load observed in the stable section. During the shield tail removal process, the internal forces undergo significant fluctuations due to changes in both load and boundary conditions, and the peak value of the axial force during the fluctuation section is eight times higher than that during the stable section, while the peak value of the bending moment during the fluctuation section is five times higher than that during the stable section. The earth and water pressure calculated using the inversion analysis method, which relies on the measured internal forces, closely matches the actual measured values. The results demonstrate that the accuracy of the water and earth pressure obtained through inversion analysis is twice as high as that obtained using the full coverage pressure method. These results can serve as a valuable reference for similar projects.

## 1. Introduction

Large-diameter shield tunnels are extensively employed in the construction of urban transportation infrastructure due to the rapid development of modern metropolitan areas. Currently, the commonly used design methods for shield tunnel segments, such as the modified idiom and beam spring models, primarily focus on stress analysis during regular operation and do not account for the impact of various factors during the construction phase. Empirical evidence from numerous shield engineering projects indicates that there is a transitional period from segment assembly into a ring to the final consolidation of both the grout and the segment, enabling them to withstand the earth’s pressure collectively. During this stage, the mechanical behavior of the segment becomes intricate, encompassing factors such as Jack thrust, grouting pressure, slurry buoyancy, formation stress, and adverse internal forces resulting from segment assembly. Hence, there is a need to conduct a systematic investigation into the mechanical behavior of segments and their vital influencing factors, considering the interactions among the shield machine, segment, and surrounding soil. Additionally, it is crucial to accurately assess the initial stress state of the tunnel structure by comprehensively understanding the laws governing tunnel structure deformation, internal forces, and external loads.

Due to the construction sequence (tunneling posture, assembly process, synchronous grouting), the conventional theoretical analysis and numerical simulation struggle to accurately depict the stress state of the shield segment during this stage. In the construction and operation of large-diameter shields, manual measurements are frequently employed to monitor tunnel deformation and changes in internal forces. Nevertheless, manual measurements are conducted using a handheld reader once or twice a day, resulting in low monitoring accuracy and susceptible monitoring results. Numerous scholars have extensively researched tunnel engineering monitoring systems. Jiang et al. [1] proposed a real-time monitoring system for surface deformation induced by shield construction in homogeneous strata, combining static-level measurements and single-point displacement meters. This system has been successfully implemented in Beijing Metro Line 14 and the new airport line. Cui and Tan [2] conducted long-term settlement monitoring for Shanghai Tunnel Line 1 since 1995, revealing that long-term service can lead to differential settlement in the tunnel’s longitudinal direction, thus causing segment disease. Huang et al. [3] developed a real-time monitoring system to analyze the interaction between cutter head excavation and surrounding rock during shield tunneling, which was applied to a tunnel project in Lanzhou, and they also proposed a back-calculation method to estimate the surrounding rock pressure. Zheng et al. [4] used optical fiber sensors to monitor the longitudinal strain, curvature, and joint deformation of segments during the tunneling of Foshan Metro Line 2. They established a segment deformation prediction model based on the beam theory, considering the variation in formation parameters. Hu et al. [5] conducted field monitoring and finite element analysis to study ground displacement, as well as longitudinal and transverse deformation of an existing tunnel when a new shield tunnel in Tianjin Metro intersected with it. The calculation results indicate minimal deformation in the lower structure of the existing tunnel. Gue et al. [6] monitored and analyzed the mechanical response of the existing tunnel structure during the process of building a new tunnel through an existing tunnel. Clayton et al. [7] used displacement sensors to monitor the settlement caused by the tunnel crossing the airport process Wang et al. [8] monitored the hoisting process of the underwater inlet and outlet of a hydraulic tunnel in a power plant and simulated the deformation of the segment joint during the lifting process. Guo et al. [9] utilized GPS and Beidou systems to monitor riverbed deformation caused by underwater shield tunneling, achieving millimeter-level monitoring accuracy. Wang et al. [10] proposed a stress analysis method for three-dimensional multilayer elastic materials, which combines domain decomposition technology and employs the generalized finite difference method to calculate the displacement and stress of subregions. The method is then compared with the finite element method and the boundary element method. The results indicate that the finite difference method exhibits high accuracy and efficiency in addressing nonlinear problems. Kabir and Aghdam [11] proposed a solution method based on the Bezier MultiStep Method to solve the nonlinear vibration and postbuckling configuration control equations of composite Euler–Bernoulli reinforced beams with graphene nanoplatelets. This method demonstrates high accuracy and robustness. Bert and Malik [12] proposed a numerical solution method, the differential quadrature method, for analyzing composite laminated plates. They utilized the first-order shear deformation plate theory to solve and verify the accuracy of the proposed method for the vibration of symmetrically laminated cross-ply plates.

In recent years, there has been a growing interest among researchers in establishing inversion analysis models based on field-monitored physical quantity data. These models aim to derive the initial parameters of the project site and propose theoretical prediction models that closely approximate the actual service state of the tunnel. Callisto and Ricci [13] utilized measured damage data from an earthquake-damaged tunnel structure in Italy. They employed the equivalent static method to perform an inversion analysis of the earthquake-induced load and validated the proposed method using a coupled dynamic finite element method that considered the interaction between the soil and structure. Fakhimi et al. [14] introduced a numerical parameter inversion analysis method. They performed a matching inversion analysis of numerical simulation results and the measured convergence value of a tunnel in Iran to determine the in situ stress and soil cohesion of the tunnel’s location. These values were then compared with the results of direct shear and plate load tests. Kim et al. [15] developed an inversion analysis method for the concrete lining ground load, considering the construction process, using convergence data from the concrete segment. Sharifzadeh et al. [16] employed a single-variable optimization algorithm to directly analyze displacement and simulate the creep characteristics of the surrounding rock in the tunnel. Lee et al. [17,18] utilized the numerical inversion analysis method to investigate the load distribution of the surrounding rock during the excavation of a multiarch tunnel. They established a correlation between the height of the load loose circle and the deformation modulus of the rock mass and derived a calculation method for the surrounding rock load in a multiarch tunnel. Yan et al. [19] presented an inversion analysis method for determining the water and soil load acting on the exterior of the tunnel. They based their method on strain data from monitored segment sections on-site and assumed a linear change in the water and soil load. The least square method was employed for inversion analysis, allowing for the calculation of the stress in the tunnel section.

The rapid development of artificial intelligence technology has led to the gradual integration of machine learning algorithms into the prediction and parameter inversion analysis processes of tunnel engineering. Ye et al. [20] proposed static leveling to monitor the real-time floating amount of segments throughout the entire process of shield tunneling. They introduced a machine learning algorithm that combines particle swarm optimization and the random forest method to predict the floating process of the segment, achieving an R2 value of 0.915. Ye et al. [21] monitored the surface settlement resulting from shield tunneling using Fiber Bragg grating sensors. They utilized the TS-BPNN algorithm to predict the subsequent settlement. Ye et al. [22] compiled a segment floating process database that incorporates geological conditions, tunnel geometry, and shield tunneling parameters. They employed a variety of machine learning algorithms to predict the floating process. Niu et al. [23] proposed an inversion analysis method that combines model testing and numerical simulation, based on the measured water and soil load distribution in a railway tunnel located in Foshan. They presented the load distribution characteristics of the tunnel segment in the soft soil layer. Vardakos et al. [24] utilized a simulated annealing algorithm and numerical simulation software to conduct an inversion analysis of the tunnel’s deformation and plastic zone. This approach has been employed in specific engineering calculations. Yu et al. [25] used a BP neural network to perform an inversion analysis of the displacement of a pile foundation. They obtained the elastic modulus, Poisson’s ratio, cohesion, and internal friction angle of a landslide. Zhao and Feng [26] employed particle swarm optimization and multilinear regression to conduct an inversion analysis of the direction and magnitude of in situ stress along the tunnel. This analysis was based on geological conditions and measured stress.

Currently, the monitoring and mechanical analysis of tunnel segment structures primarily concentrate on the operational stage after tunnel construction is completed, with less consideration given to the mechanical behavior of shield tunnel segments during construction processes. Additionally, the soil pressure acting on the segment significantly impacts segment design, particularly for large-diameter underwater shield tunnels. A notable disparity exists between the current segment design theory and the actual stress state, making it challenging to ascertain whether the existing segment design load method can ensure the safety and reliability of the segment during both construction and operation. The development of deep learning technology and the proposal of inversion analysis theory offer potential solutions to this issue.

This paper establishes an on-site monitoring system for the Internet of Things using embedded optical fiber sensors. The variation in water and earth pressure and mechanical response law of the segment ring in the stage of shield assembly and removal from the shield tail are analyzed. A Bayesian-genetic algorithm inversion analysis model of water and earth pressure was proposed. Specifically, the optimization objective function for inversion analysis is developed by modifying the idiom, which establishes a relationship between the segment’s internal force and external load. The measured internal force data are then analyzed using a genetic algorithm. Additionally, a Bayesian algorithm is employed to optimize various parameters, such as population number, gene length, crossover probability, and mutation probability, within the genetic algorithm. This optimization aims to enhance computational efficiency and accuracy. Finally, the inversion analysis load is compared with the field-measured value, and recommendations regarding the load values are provided.

## 2. Project Overview

### 2.1. Project Background

**Figure 1:**
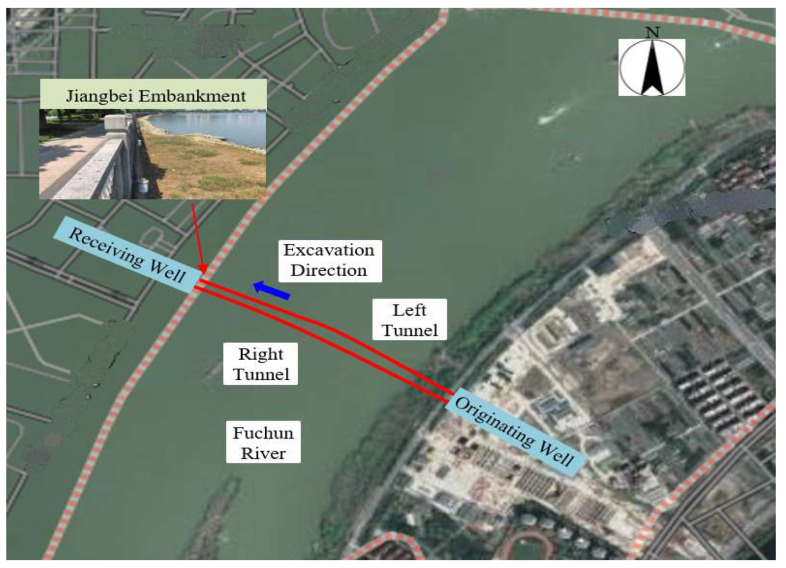
Location of the Qinwang tunnel.

The Qinwang tunnel is situated 3 km downstream from the Fuyang bridge. This tunnel is the first road–rail tunnel in Zhejiang Province of China. It spans a total length of 3066 m, with 2868 m comprising the main tunnel and 1254 m forming the shield length in the river crossing section, as shown in Figure 1. In the river crossing section, a double-tube circular shield is employed. The shield cutter head has an outer diameter of 15.8 m, while the shield tunnel has an outer diameter of 15.2 m and an inner diameter of 13.9 m. The segment is constructed of C60 reinforced concrete and takes the form of a flat plate structure. It incorporates a universal wedge ring with a wedge amount of 52 mm, has a ring width of 2 m, a segment thickness of 0.65 m, and employs a main reinforcement HRB400e with a diameter of 28 mm. The outer main reinforcement of the segment has a protective layer thickness of 0.05 m, while the inner main reinforcement has a protective layer thickness of 0.04 m. Staggered assembly is achieved using inclined bolt connections, and the tunnel is constructed using the 7 (standard block) + 2 (connecting block) + 1 (key block) block mode.

**Figure 2:**
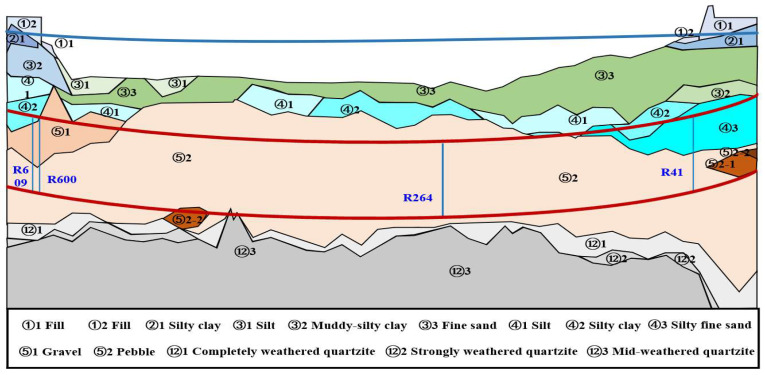
Geological section and monitoring point settings of Qinwang tunnel.

The Qinwang tunnel predominantly traverses a stratum of pebble soil, characterized by uneven particle distribution, and challenges in transmitting force between particles. This soil layer is a classic example of mechanical instability. The overlying soil layer predominantly consists of silty clay, silt, and fine sand, exhibiting strong permeability. The riverbed elevation above the Qinwang tunnel typically ranges from −5.43 m to 3.64 m. In these geological conditions, the operation of the Qinwang tunnel is primarily susceptible to risks including uneven settlement deformation, segment cracking, and water seepage. Consequently, the design of the Qinwang tunnel employed the beam–spring model for calculations, incorporating earth and water separation in load calculation. Simultaneously, a safety factor of 1.25 was considered for structural anti-floating verification. The specific geological parameters are presented in Figure 2.

### 2.2. Design and Implementation of Monitoring System

**Figure 3:**
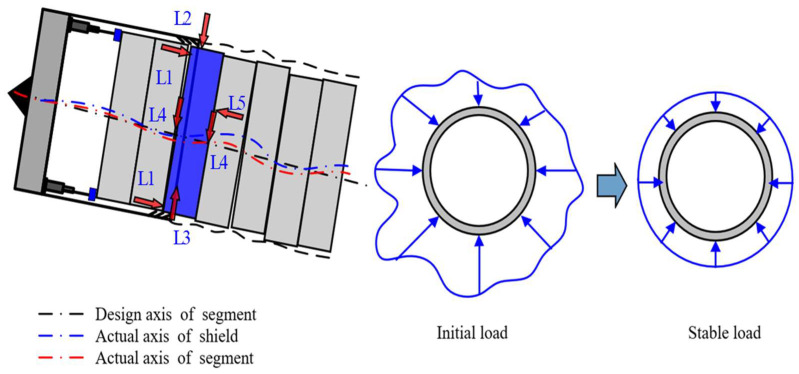
Schematic illustration of load variation.

To investigate the mechanical behavior and key influencing factors of the segment under the interaction between the shield machine and the surrounding soil, it is necessary to analyze the stress state of the shield machine during tunneling. The primary sources of mechanical behavior in the shield machine segment can be attributed to four factors, as shown in Figure 3:

Jacking force L1: During the construction of a shield tunnel, each jack of the shield machine exerts force on the segment through a pallet, which divides the cross-section into different areas. However, the thrust in each area varies during excavation and assembly. Measured data indicates that the thrust at the bottom of the tunnel arch can be twice as high as the thrust at the top of the tunnel arch [27].

Shield tail brush force L2: The shield tail brush, typically made of steel wire, is installed at the rear of the shield shell of the shield machine. During synchronous grouting of the shield machine, the shield tail seal is employed to prevent slurry from flowing back into the shield machine. As the tunneling distance increases, the shield tail brush becomes enveloped by hardened slurry, resulting in increased stiffness and generating force reactions on the segment.

Shield machine shell force L3: Due to the influence of the tunnel design axis during the tunneling process, the shield machine may not always align perfectly parallel to the segment. This misalignment is particularly notable on uphill and downhill slopes, where the segment may meet the shield shell of the shield machine, resulting in a force exerted on the segment.

Friction force L4 and extrusion force L5 of adjacent segment: When the segment is assembled, they are subject to boundary conditions imposed by the adjacent segment. Longitudinally, the adjacent segment exerts an extrusion force on the assembled segment, while laterally, a frictional force is generated.

Throughout the shield tunnel construction, the evolving construction process leads to gradual changes in the acting forces L1~L5, delineated into three phases: Phase 1 involves the segment being situated within the shield shell, where the jack thrust L1 directly influences the segment, inducing longitudinal stress fluctuations. Phase 2: The segment is gradually removed from the shield tail. The brush force L2 and shield shell force L3 act on the outer side of the segment, causing internal stress fluctuations in the segment along with the jack thrust L1. Phase 3: The segment dislodges from the shield tail. The L1~L3 forces gradually dissipate, and the stress on the segment gradually balances under the action of earth and water pressure. In the three phases, both the frictional force L4 and the extrusion force L5 act on the segment, affecting the stress fluctuations. Consequently, the monitoring system design involves placing an earth pressure gauge between the segment to monitor L1 and L5. Additionally, an earth pressure gauge and seepage pressure gauge are positioned outside the segment to monitor L2 and L3. An earth pressure gauge is positioned between blocks to monitor L4.

**Figure 4:**
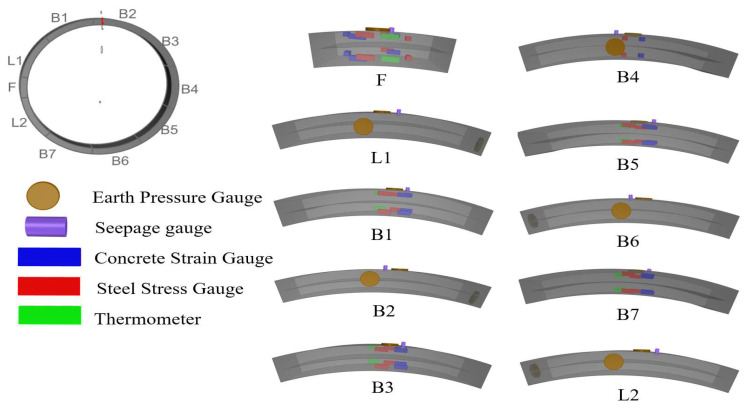
Schematic illustration of embedded sensor.

The experimental ring is buried at the left and right tunnel rings, R41, R264, R600, and R609. To achieve monitoring objectives, earth pressure gauges and seepage pressure gauges are positioned on the outer side of each segment to track changes in the earth and water pressures. Concrete strain gauges and steel stress gauges are also installed on each segment to monitor changes in internal forces. Additionally, upon completion of segment assembly, a bolt stress gauge is installed at a designated location to monitor changes in bolt stress during shield tunneling construction. This study focuses on the R600, providing a detailed introduction to the design, installation, and data acquisition process of the on-site Internet of Things monitoring system. Furthermore, it analyzes the mechanical behavior of an ultra-large-diameter shield tunnel segment during the construction stage. The division of blocks and the distribution of embedded sensors in the test ring segment are illustrated in Figure 4.

The resolution and accuracy of sensors are shown in Table 1.

**Figure 5:**
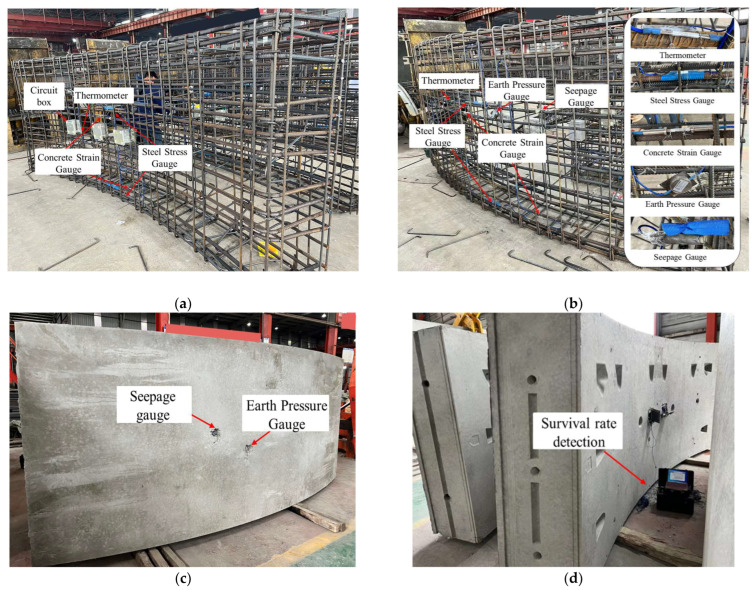
Installation and pouring of embedded sensors: (**a**) embedded sensor inside segment; (**b**) embedded sensor outside the segment; (**c**) segment pouring completed; (**d**) sensor survivability detection.

All sensors are embedded in the segment factory according to the design specifications, as shown in Figure 5. Fiber Bragg grating sensors are interconnected using optical cables, which converge to the concealed box in a series configuration. To ensure data quality, both ends of the optical cable are connected to the concealed box, with 16 m of excess length reserved at one end and 5 m reserved at the other end for backup purposes. The osmometer cable is also connected to the concealed box. Once the segment casting and curing are completed, the concrete covering the earth pressure gauge and osmometer is carefully chiseled off. A handheld demodulator is then used to collect the wavelength and frequency readings from the sensor, which are compared with the preset values to verify the reliability of the sensor during the monitoring process.

The segment design wedge reaches its maximum at block B4 and its minimum at block F. Therefore, during the segment assembly process, B4 blocks are first assembled, followed by the left and right blocks, and finally, the F blocks are assembled. To ensure the collection of necessary data during the shield construction process, the demodulator and distribution box are installed at the designated position of the tunnel arch waist (R556 R557) one day in advance.

**Figure 6:**
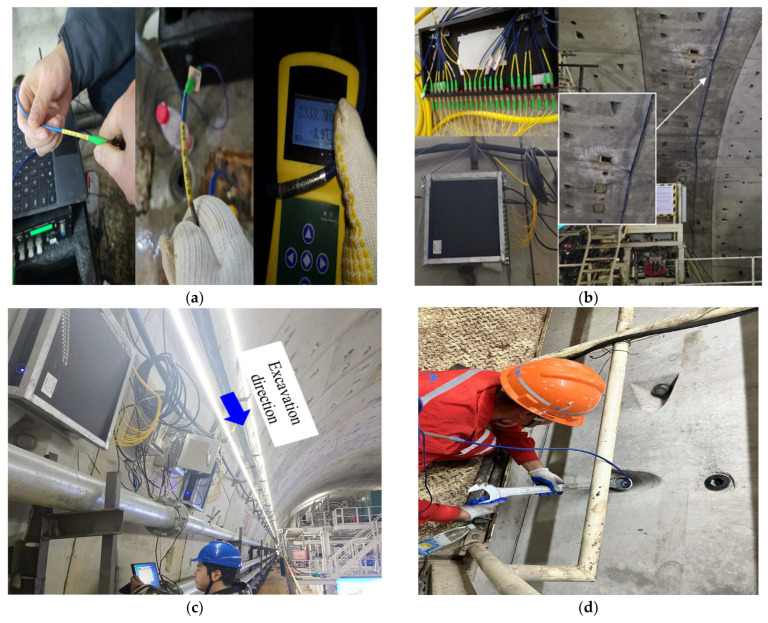
On-site monitoring system installation of Internet of Things: (**a**) sensor initial value reading; (**b**) data aggregation and transmission; (**c**) real-time data check; (**d**) installation of bolt stress gauge.

For the installation of the left half-ring instrument and demodulator, testers 1 and 2 connect the embedded sensors in the left half-ring to the demodulator using collection cables in blocks. In this process, priority is given to connecting the earth pressure gauges between the rings to monitor the influence of Jack thrust on the stress of the segment ring. After the installation of the embedded sensors, testers 1 and 2 install the bolt stress meter and connect its cables to the demodulator in blocks. Meanwhile, testers 3 and 4 use a handheld reader to collect initial values from the sensors at the left waist of the tunnel, which are then connected to the flange plate. The flange plate is further connected to the demodulator to enable automatic data collection, with an acquisition frequency of 1 s per time. Additionally, tester 6 is assigned to check the demodulator data in real time. Testers 4 and 5 follow a similar procedure to assemble the right half-ring instrument and demodulator to the left flange, as shown in Figure 6.

## 3. Monitoring Results

### 3.1. Shield Tunneling Parameters

**Figure 7:**
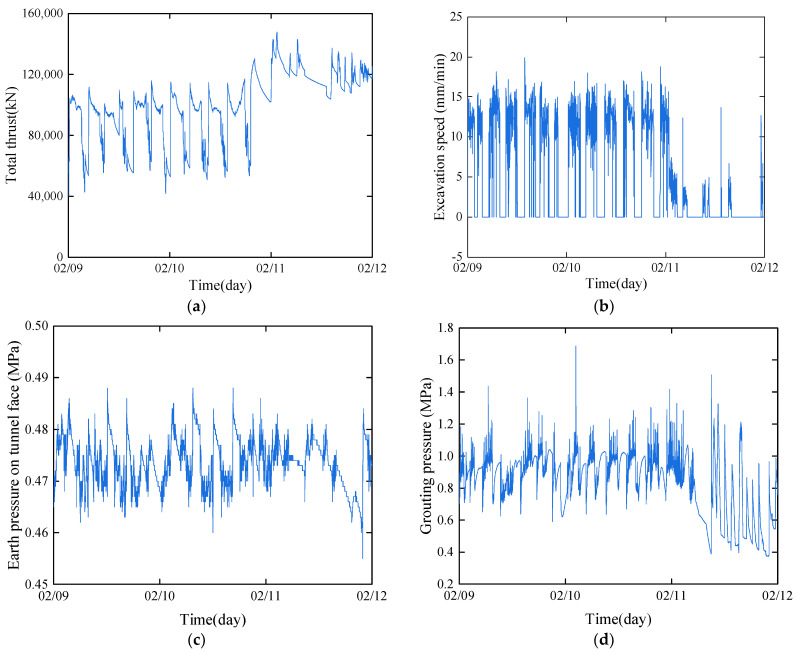
Shield tunneling parameters: (**a**) total thrust; (**b**) excavation speed; (**c**) earth pressure on tunnel face; (**d**) grouting pressure.

The data for shield tunneling were collected from the Herrenknecht slurry shield used in the left line of the Qinwang tunnel project. The acquisition frequency was set at 1 min/time. The cutter head excavation diameter is 15.80 m, the outer diameter of the shield tail is 15.67 m, and the distance from the segment assembler to the shield tail is approximately 7 m, which is 2.5 times the width of the segment ring. To prevent slurry from flowing into the front of the shield and front slurry from affecting the grouting effect by flowing into the gap at the shield tail, four sealing brushes and a grout stop plate are installed at the tail of the shield. The test ring (R600) shield began driving at 13:00 on 9 February 2023, completed driving at 16:31, and finished assembly at 18:10. The total thrust, tunneling speed, earth pressure, and grouting pressure of the tunnel face during the tunneling from the R600 to the R611 shield are presented in Figure 7. At 0:16 on 12 February 2023, the cutter head was replaced. The total thrust value of the shield remained relatively stable between 40,000 kN and 120,000 kN, compared with the designed maximum thrust of 228,004 kN. The tunneling speed varied between 0–15 mm/min. A propulsion speed of 0 for an extended period (over 30 min) indicates that the segment is in the assembled state. It can be observed that during the driving of the 600–611 ring, the driving parameters exhibited slight fluctuations.

### 3.2. Water and Earth Pressure

**Figure 8:**
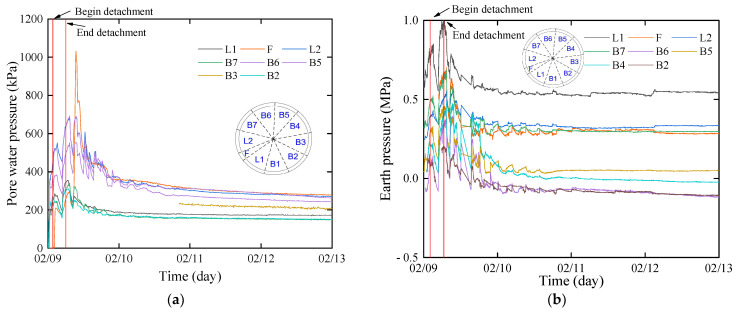
Water and earth pressure outside the test segment: (**a**) outside water pressure; (**b**) outside earth pressure.

During the process of segment removal from the shield tail, the segment is influenced by various factors, including jack thrust, shield tail brush force, shield shell force, and grouting pressure, presenting a challenge to the structural safety of the segment. Excessive jack thrust leads to segment damage [28], while an excessive shield shell force results in segment misalignment [29]. The variation curve of water and earth pressure during the segment’s removal from the shield tail is depicted in Figure 8.

Based on the shield machine’s tail length data, the test ring should begin to be removed from the shield tail at the midpoint of the R602 (9 February 2023 23:41) and be completely removed from the shield tail at the midpoint of the R603 (10 February 2023 4:08). During the removal of the segment, the total pressure outside the segment undergoes significant fluctuations, with the peak pressure reaching 1 MPa. The total pressure of each segment ring is approximately twice the pressure in the stable stage. Eventually, the total pressure outside the segment stabilizes around 11 February 2023 18:00, with a stable value of about 0.4 MPa.

Before the segment’s removal, the external pressure of the segment measured by the osmometer was approximately 300 kPa, which may be attributed to the influence of seal grease at the shield tail. During the stage of segment removal, the pore water pressure fluctuated due to the influence of segment assembly, with the declining period corresponding to the assembly of the R603. After the complete removal of the shield tail, under the effect of grouting pressure, the external water pressure rapidly increased, peaking at 1032 kPa, and then gradually dissipated. The water pressure stabilized around 11 February 2023 16:00, ranging between 155 kPa and 300 kPa, which is close to the calculated hydrostatic pressure (170.62 kPa).

During the process of the segment being removed from the shield shell, the jack force thrust, shield tail brush force, and shield shell force induce pressure fluctuations on the outer side of the segment. With the advancement of the shield tunneling machine, these forces gradually diminish, leading to a gradual stabilization of pressure on the outer side of the segment. Consequently, the process of pressure fluctuations on the outer side of the segment reaching a steady state is a dynamic process that continuously evolves with the external forces acting on the segment.

### 3.3. Stress between Blocks

**Figure 9:**
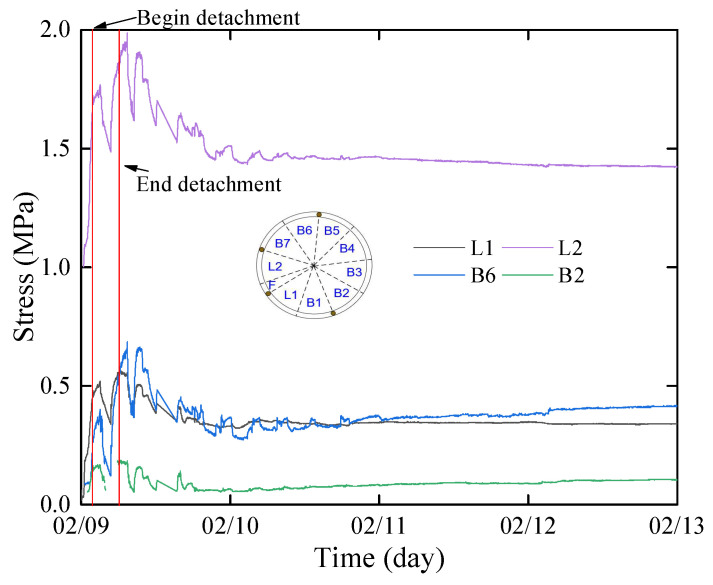
Stress between blocks.

During the segment removal process in the construction period, the forces are temporarily balanced through the action of Jack thrust, friction between segment blocks and rings, and bolt preload. As the shield machine advances, the relative position and contact relationship between segments constantly change, and the stress between segments is also adjusted to rebalance [30]. The measured stress between segments is depicted in Figure 9 during the segment removal of the shield tail. Apart from block L2, the peak stress ranges between 0.3~0.7 MPa and gradually stabilizes as the shield machine moves away. The elevated stress value in the L2 block can be attributed to the higher jack thrust in that area compared with others.

### 3.4. Stress between Segments

**Figure 10:**
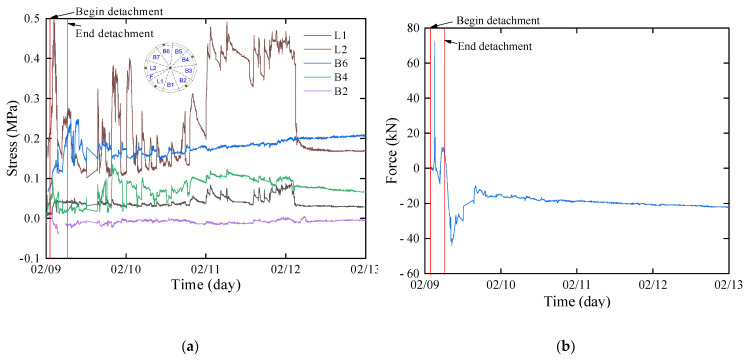
Stress between segment and bolts: (**a**) stress between rings; (**b**) bolt bearing force.

After the completion of segment removal during the construction period, the main factors affecting the system are the jack thrust and the squeezing force between adjacent segments. The jack thrust gradually dissipates as the shield machine moves away. The changes in stress between segment rings are illustrated in Figure 10a. Compared with Figure 7, it can be observed that the stress between rings fluctuates periodically. It is primarily caused by the cyclic loading of the jack, resulting in longitudinal extrusion and release of the segment ring. The stress gradually stabilizes after 3 days. By this time, the shield machine has advanced to the R611, approximately 20 m away from the test ring. The stress of the longitudinal bolts reflects the dissipation of stress between segment rings. The stress of the L2 circumferential bolt is depicted in Figure 10b, reaching a peak value of 72 kN during the shield tail extraction stage. It fluctuates during subsequent segment advancement and assembly processes and gradually stabilizes.

### 3.5. Internal Force of Segment

Based on strain measurements of the segment concrete, it is possible to calculate the bending moment and axial force at various positions of the segment ring using elementary beam theory. The calculation formula for this is as follows:

(1) $$\varepsilon_{i}=\frac{\sigma_{i}}{E_{c}}$$

(2) $$N=\frac{bh}{2}\left(\sigma_{1}+\sigma_{2}\right)$$

(3) $$M=\frac{E_{c}bh^{2}}{12}\left(\sigma_{1}-\sigma_{2}\right)$$

where $E_{c}$ is the elastic modulus of concrete; h is the segment thickness; b is the ring width of the segment; N is the circumferential axial force of the segment; and M is the circumferential bending moment of the segment.

**Figure 11:**
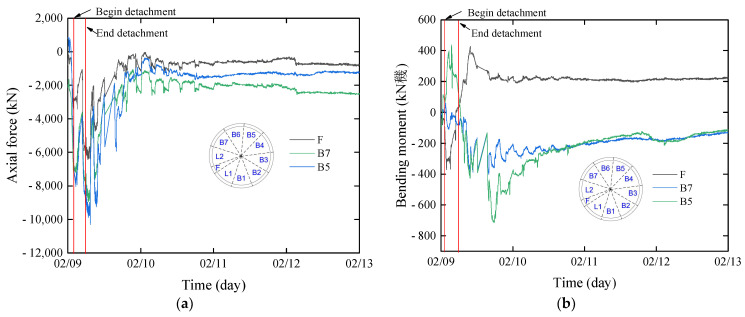
Internal force of segment: (**a**) axial force; (**b**) bending moment.

During the process of segment removal from the shield tail, the internal force fluctuation can be categorized into three stages: preremoval fluctuation, removal phase fluctuation, and postremoval stabilization as shown in Figure 11. The direction of internal forces is defined in accordance with Lin et al., where axial force is considered positive when the segment is compressed, negative for tension, and positive for bending moment when the inner side is compressed, while negative for tension. Taking the internal force of block B5 as an example, prior to removal from the shield tail, the axial force varies between 798.23 kN~−6491.89 kN, and the bending moment ranges −86.48 kN·m~115.96 kN·m. The positive axial force is attributed to the dragging effect during the adjacent the segment removal from the shield tail, while the shift from positive to negative before removal is due to the jack thrust applied to the segment, causing the shield machine to advance and exert pressure on the segment. The transition of the bending moment from inner tension to inner compression is a result of this force change. During the detachment process, the axial force varies between −90,210.75 kN and −3881.80 kN, and the bending moment ranges from 77.43 kN·m to 437.41 kN·m. The fluctuation during this stage occurs as the segment gradually separates from the shield tail, the dissipation of the jack thrust. However, factors such as the shield shell force and shield tail brush force result in negative axial force and positive bending moments of the segment. After the shield tail removal, the fluctuation tends to stabilize, with the axial force varying between −10,314.35 kN~−364.98 kN and the bending moment ranging from 126.766 kN·m~−713.46 kN·m. The removal of the shield tail and the grouting pressure being applied to the outer side of the segment, lead to significant fluctuations in the axial force and bending moment of the segment. As the grouting pressure dissipates and earth and water loads are applied in the segment, it gradually leads to the stabilization of the bending moment and internal force of the segment.

## 4. Inversion Analysis of Load

The earth pressure exerted on the segment significantly influences its design theory, making it crucial to establish a calculation method for segment earth pressure that aligns with actual shield tunnel engineering practices. Presently, the full coverage pressure theory, utilized in segment design, relies on numerous assumptions, such as empirical values for the lateral soil pressure coefficient. These assumptions deviate significantly from the actual stress state, raising doubts about the existing design load method’s ability to ensure the safety and reliability of the segment during construction and operation. Addressing these concerns, this study is based on the easily measurable internal force data of the segment, in combination with a Bayesian-genetic algorithm, to develop an inversion analysis method for determining external earth pressure on the segment.

### 4.1. Objective Function of Inversion Analysis

The load structure is a widely employed approach in the design of shield tunnel segments. This method assumes that the internal forces of the segment result from the linear superposition of internal forces caused by earth water pressure, lateral soil water pressure, self-weight, and formation reaction on the segment. The burial depth of the experimental crown is 22.08 m, and the groundwater depth at the crown is 17.4 m, the geological parameters of the test segment are shown in Table 2.

The calculation formula, as presented in Table 3, considers the load as a symmetric load and provides a calculation method for the internal forces within the 0~π. The remaining half of the internal force distribution can be obtained through symmetry [13,31].

Based on the stress load inversion method proposed by Feng et al. [32] and Zhong et al. [33], the determination coefficient is utilized as the objective function for calculating the inversion load of internal forces. This study adopts their approach for setting the objective function and employs the stress inversion analysis method. The objective function is defined as follows:

(4) $$F\left(X\right)=\beta_{1}\cdot M\left(X\right)+\beta_{2}\cdot N\left(X\right)$$

(5) $$M\left(X\right)=1-\frac{{\sum_{i=1}^{n}\left(M_{i}-{\hat{M}}_{i}\right)}^{2}}{{\sum_{i=1}^{n}\left(M_{i}-\bar{M}\right)}^{2}}$$

(6) $$N\left(X\right)=1-\frac{{\sum_{i=1}^{n}\left(N_{i}-{\hat{N}}_{i}\right)}^{2}}{{\sum_{i=1}^{n}\left(N_{i}-\bar{N}\right)}^{2}}$$

where $X=\left\{\left.x_{1},x_{2},x_{3}\ldots ,x_{k}\right\}\right.$ are the parameters to be solved; $\beta_{1},\beta_{2}$ is the weighted coefficient; M is the calculated value of bending moment; $\hat{M}$ is the measured value of bending moment; $\bar{M}$ is the average value of the measured value of bending moment; N is the calculated value of axial force; $\hat{N}$ is the measured value of axial force; and $\bar{N}$ is the average value of measured value of axial force.

**Figure 12:**
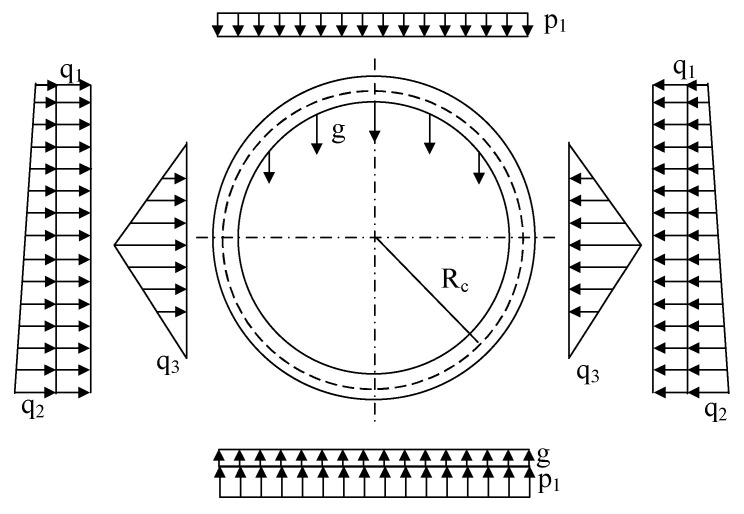
Distribution pattern of load on segments.

This study employs the revised load structure model as the load pattern for load inversion, as depicted in Figure 12. The model assumes a uniformly distributed rectangular distribution mode for soil and water pressure $p_{1}$ on the lining, a uniformly distributed trapezoidal distribution mode for lateral soil and water pressure $q_{1}$ and $q_{2}$, and a uniformly distributed triangular distribution mode for horizontal formation resistance $q_{3}$. Assuming the parameter $p_{s},p_{w},\lambda ,k$ is requires inversion analysis, the calculation expressions for each load are as follows:

(7) $$\left\{\begin{array}{l} p_{1}=p_{s}+p_{w}\\ q_{1}=k_{s}(p_{s}+\gamma t/2)+p_{w}+\gamma_{w}t/2\\ q_{2}=2R_{c}\left(k_{s}\gamma +\gamma_{w}\right)\\ q_{3}=k_{r}\delta \end{array}\right.$$

where $p_{s}$ is the earth pressure of the tunnel crown; $p_{w}$ is the water pressure of the tunnel crown; $k_{s}$ is the lateral pressure coefficient; t is the thickness of the segment γ is the density of the soil; $\gamma_{w}$ is the density of the water; $R_{c}$ is the calculated diameter of the tunnel; $k_{r}$ is the resistance coefficient of the soil; and δ is the horizontal displacement of the segment.

### 4.2. Bayesian-Genetic Algorithm Inversion Analysis Model

The genetic algorithm is an optimization algorithm that simulates the selection and mutation phenomenon in biological genetic systems [34,35,36]. It begins with a random initial population and performs random selection, crossover, and mutation operations. The obtained value is compared and replaced with the target value to obtain the optimal solution for the problem. However, the genetic algorithm also has limitations. For instance, during the process of setting superparameters, if the population size is small, the computation may prematurely converge, resulting in a local optimal value rather than the global optimal value of the model. Conversely, if the population size is too large, it requires large-scale parallel computation, leading to high computational costs and impacting efficiency. Therefore, selecting the superparameters of the genetic algorithm in a reasonable manner to ensure both effective computation and reasonable calculation time is a significant technical challenge in utilizing the genetic algorithm.

Bayesian optimization is an algorithm that incorporates previous parameter information to update the prior function and continually adds sample points to enhance the posterior distribution of the objective function [37,38,39]. This is achieved through the Gaussian process, allowing for improved adjustment of the current parameters.

In this study, the Bayesian algorithm is employed to optimize the population number, gene length, crossover probability, and mutation probability within the genetic algorithm. Specifically, the Bayesian algorithm randomly generates various combinations of superparameters, which are then inputted into the genetic algorithm model. Each set of superparameters returns a fitness value for the objective function, which serves as the optimization objective for the Bayesian algorithm in inverse optimization.

### 4.3. Result of Inversion Analysis

The load values of the inversion analysis are shown in Table 4. According to the inversion analysis results, the coefficient of determination (R^2^) for earth pressure is determined to be 0.8213. The inversion analysis value is found to be 76.21% higher than the measured value, while the calculated value based on full coverage pressure theory is 153.04% higher than the measured value. This difference can be attributed to the arching effect of the earth pressure above the test ring, which causes the measured earth pressure to be lower than the earth pressure calculated using the theory of the full coverage pressure. Regarding water pressure, the inversion analysis value is 17.86% higher than the measured value, whereas the calculated value based on full coverage pressure theory is 5.58% higher than the measured value. This discrepancy may be related to the setting of the proportional coefficient of axial force and bending moment in the earth pressure backcalculation model.

Employing root mean square error (RMSE) as the evaluation index for earth pressure calculation, the discrepancy between the calculated value using the inversion algorithm and the true value is 124.95. In contrast, using the full coverage pressure theory yields a discrepancy of 255.84. The inversion algorithm exhibits a 51.16% reduction in calculation error compared with the full coverage pressure theory. Currently, the accuracy of the load inversion model proposed by our study institute is not very satisfactory due to limited measured data. In our next research, we will explore data augmentation methods to enhance the model’s accuracy.

**Figure 13:**
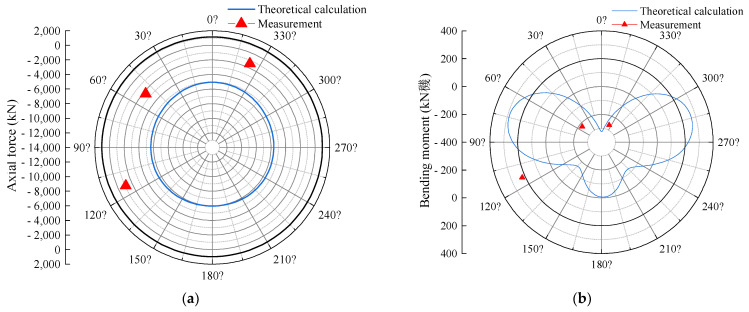
Distribution pattern of load on segment: (**a**) axial force; (**b**) bending moment.

The internal forces of the segment are calculated based on the retrieved water and earth pressure from the given parameters, as shown in Figure 13. The results indicate a satisfactory agreement between the calculated and measured values of the bending moment. However, there is a significant disparity between the calculated and measured values of the axial force. This discrepancy arises from the fact that the bending moment is predominantly influenced by the transverse load, whereas the axial force is affected by both the longitudinal jacking thrust and the friction between the segments. The utilization of a two-dimensional theory in the calculation fails to capture the three-dimensional mechanical effects of the segment, leading to a substantial error.

## 5. Conclusions

This paper presents a study conducted on a super-large-diameter highway–rail combined shield tunnel project. An on-site monitoring system based on the Internet of Things (IoT) is established to continuously monitor the water and soil loads, as well as internal forces during the process of segment assembly and removal from the shield tail. The measured results are then utilized to invert the water and soil loads, leading to the following main conclusions:

(1) The earth and water pressure on the outer side of the segment undergo two stages of fluctuation and stabilization. Once the segment is removed from the shield tail, it undergoes dynamic adjustments due to the combined effects of the longitudinal component of the jack thrust, brush force on the shield tail, shield shell force, formation pressure, grouting pressure, and segment friction generated by adjacent assembled segments. Eventually, the loads gradually stabilize. The peak load during the fluctuation stage is twice the load observed during the stable stage. Greater initial internal forces will significantly influence forces during tunnel operation. Excessive earth and water pressure in the fluctuating section can lead to segment damage and seepage, ultimately impacting the tunnel’s long-term functionality and robustness.

(2) After the segment is removed from the shield tail, the internal forces experience violent fluctuations before reaching a state of stability. The peak value of the axial force during the fluctuation stage is eight times higher than the stable value. This fluctuation is primarily attributed to changes in boundary conditions (such as jack thrust and extrusion pressure on the front and rear segment) during the removal process. Similarly, the peak value of the bending moment during the fluctuation stage is five times higher than the stable value, mainly due to variations in load conditions (shield machine force, grouting pressure, and water and soil pressure) during the segment ring extraction from the shield tail.

(3) To invert the water and soil loads on the outer side of the segment, a Bayesian genetic algorithm is proposed. The obtained earth pressure and water pressure values exhibit errors of 76.21% and 17.86%, respectively, when compared with the measured values. The inversion algorithm exhibits a 51.16% reduction in calculation error compared with the full coverage pressure theory, indicating that the proposed algorithm provides results closer to the actual measurements.

## Figures and Tables

**Table 1:** Resolution and accuracy of sensors.

Measuring Content	Sensors	Range	Resolution	Accuracy
Earth pressure and segment pressure	Earth pressure gauge	0~2000 kPa	1 kPa	10 kPa
Concrete stress	Concrete strain gauge	−1500~1500 με	0.1 με	±1 με
Steel stress	Steel stress gauge	0~400 MPa	2 kPa	10 kPa
Internal force of bolts	Bolt stress gauge	0~550 kN	0.5 kN	3 kN
Pore water pressure	Seepage gauge	0~1000 kPa	0.25 kPa	1 kPa

**Table 2:** Geological Parameters.

Soil	Unit Weight (kN/m^2^)	Cohesion (kPa)	Friction (°)	Earth Pressure Coefficient	Thickness (m)
Fill	19	10	10	0.6	5.25
Muddy–silty clay	18.3	14	13.2	0.7	7.12
Silt	19.2	16.25	16.98	0.5	3.35
Silty clay	19.6	31.34	16.45	0.55	4.4
Gravel	20	0	31	0.35	1.96
Pebble	20	0	31	0.35	8.2

**Table 3:** Internal force calculation.

Load Type	Calculation Range	$M_{\alpha }\left(\mathrm{kN}\cdot m\right)$	$N_{\alpha }\left(\mathrm{kN}\right)$
$p_{1}$	0~π	$\frac{1}{4}P_{1}R^{2}\left(1-2\sin^{2}\alpha \right)$	$P_{1}R\sin^{2}\alpha$
$q_{1}$	0~π	$\frac{1}{4}P_{2}R^{2}\left(1-2\cos^{2}\alpha \right)$	$P_{1}R\cos^{2}\alpha$
$q_{2}$	0~π	$\frac{1}{48}R^{2}\left(\begin{array}{l} 6+3\cos\left(\alpha \right)-12\cos^{2}\left(\alpha \right)\\ -4\cos^{3}\left(\alpha \right) \end{array}\right)$	$\frac{1}{16}R\left(\begin{array}{l} -\cos\left(\alpha \right)+8\cos^{2}\left(\alpha \right)\\ +4\cos^{3}\left(\alpha \right) \end{array}\right)$
$q_{3}$	$0\sim \frac{\pi }{4}$	$k_{r}\delta R^{2}\left(0.2346-0.3536\cos\alpha \right)$	$0.3636k_{r}\delta R\cos\alpha$
	$0\sim \frac{\pi }{2}$	$k_{r}\delta R^{2}\left(\begin{array}{l} -0.3487+0.5\sin^{2}\alpha \\ +0.2357\cos^{3}\alpha \end{array}\right)$	$k\delta R\left(\begin{array}{l} -0.7071\cos\alpha +\cos^{2}\alpha \\ +0.7071\sin^{2}\alpha \cos\alpha \end{array}\right)$
g	$0\sim \frac{\pi }{2}$	$gR^{2}\left(\begin{array}{l} -\pi \sin\alpha +\left(\pi -\alpha \right)\sin\alpha \\ +\pi \sin^{2}\alpha +1/6\cos\alpha \end{array}\right)$	$gR\left(\begin{array}{l} -\pi \sin\alpha +\left(\pi -\alpha \right)\sin\alpha \\ +\pi \sin^{2}\alpha +1/6\cos\alpha \end{array}\right)$
	$\frac{\pi }{2}\sim \pi$	$gR^{2}\left(\begin{array}{l} 3/8\pi -\left(\pi -\alpha \right)\sin\alpha \\ +5/6\cos\alpha \end{array}\right)$	$gR^{2}\left(\begin{array}{l} \left(\pi -\alpha \right)\sin\alpha +\\ 1/6\cos\alpha \end{array}\right)$

**Table 4:** Result of inversion analysis.

	$p_{s}$	$p_{w}$	$k_{s}$	$k_{r}$
Measured	163.96	161.58	/	/
Theoretical	419.80	170.61	/	/
Inversion analysis value	288.91	190.44	0.46	1217.89

## Data Availability

The data that support the findings of this study are available from the corresponding author upon reasonable request.
